# Elderly suicides in India: an emerging concern during COVID-19 pandemic

**DOI:** 10.1017/S1041610220001052

**Published:** 2020-06-03

**Authors:** Usha Rana

**Affiliations:** Dept. of Sociology and Social work, Dr. Harisingh Gour Central University, Sagar 470003, Madhya Pradesh, India

The whole world has had encounters with the unbidden COVID-19 disease since November 2019. Due to the nonexistence of vaccination, the infection is rapidly spreading in the world. A recent study has highlighted the adverse effect of COVID-19 on mental health with depressive and anxious symptomatology from moderate to severe among the general public (Wang *et al.*, 2020), indicating the ubiquitous consequences of precariousness and health-related fears. However, it is required to further investigate beyond the population level to comprehend the disordering of lives and routines at a personalized level as a repercussion of COVID-19 and its pessimistic psychological ramifications. The WHO has recommended mandatory self-isolation for older adults from the society. However, social isolation of the elderly population has intensified other concerns, including neurocognitive, autoimmune, cardiovascular, and mental health, which is referred to as “serious public health concern”. Furthermore, social disconnectedness causes depression and anxiety in older adults (Santini *et al.*, 2020).

In India, more than 300 suicides were reported during the lockdown as “non-coronavirus deaths” due to mental torment. According to the data, 80 people killed themselves due to the fear of being infected and loneliness. This emerging situation puts the mental health of the elderly at higher risk of relapse as they are already susceptible to melancholy and disquietude (Flint *et al.*, 2020). Furthermore, elderly those are living alone find themselves unprotected due to the lack of social support in the present scenario. Out of a total of 8.6% elderly population, approximately 29% of elderly persons are residing in urban areas. Fifty-three million older adults are forced to live in poverty and are struggling against financial insecurities, non-availability of essential groceries, inaccessibility of technology, and lack of socialization resources (Cohen-Mansfield *et al.*, 2016). A study estimates that 6% of elderly citizens live alone in India. Further, 10%–20% of them are enduring from mental desolation and loneliness.

In this study, I delineate the case study of five older adults who committed suicide due to a relapse of depressive disorder. An elderly couple from Punjab state have ended their life by consuming a poisonous substance under the scare of the COVID-19 outbreak (*Hindustan Times*, 2020). A suicide note was recovered in which they mentioned “We are finishing our lives. No one is responsible for this. There has been a tension due to coronavirus. We both were also ill”. Another suicide case of the elderly was reported from Maharashtra state, where a 75-year-old adult hanged himself from a ceiling fan in his residence (*The Times of India*, 2020). The police officers found a suicide note which solely mentioned only two words, “corona fear”. A similar case of suicide of 60-year-old adult was reported from Tamil Nadu state who hanged himself from a window grill in the isolation ward of a government hospital out of corona scare (*The Indian Express*, 2020). The officials said, “His samples tested negative for COVID-19. The result has come just now”. Due to fear of corona, another suicide case was reported from Punjab state, where a 65-year-old woman committed suicide, citing concern that she would infect her daughters (*The Tribune*, 2020).

**Figure 1.**
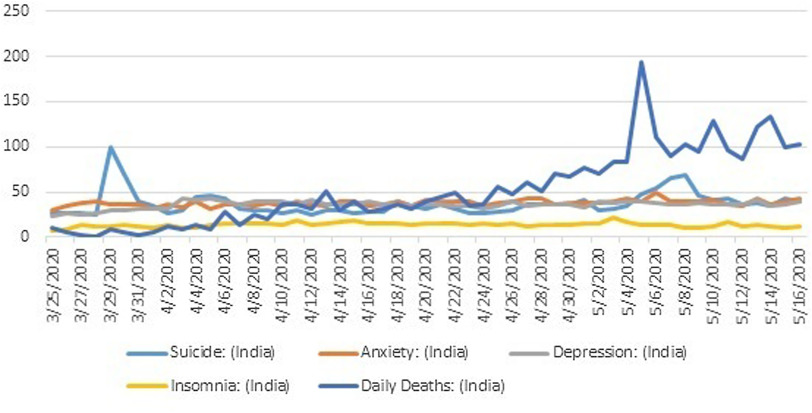
Graphical representation of RSVs on google trends throughout India.

These cases described here explore that elderly, who are already suffering from mental disorders, are more vulnerable to COVID-19 pandemic, and the social consequences of COVID-19 have invigorated them to end their lives. The excessive information about consequences of COVID-19 for the elderly proclaimed by the news channels and social media led to the development of initial anxiety. Recently, google trends of relative search volumes (RSVs) associated with mental health corroborate this study of the adverse effects on the mental health of the elderly. I conducted analysis and found a correlation between the daily COVID-19 deaths in India and the following keywords: “depression”, “anxiety”, “insomnia”, and ‘’suicide”. The RSV data were collected for the period between March 25, 2020, and May 16, 2020, as shown in Figure 1. The investigations of the Pearson correlation coefficient found a significant positive correlation between the daily COVID-19 deaths reported and the RSVs for “depression” (r = 0.3611, p < 0.05), “anxiety” (r = 0.5053, p < 0.05), “suicide” (r = 0.2004, p < 0.05), and “insomnia” (r = 0.0984, p < 0.05). This is obvious because a 40% increment is reported in the cases of mental health issues in the last 52 days in India. These findings suggest that family interventions with social cohesion may lead to improving the mental health of the elderly, which can be referred to as a phenomenon of resilience. However, the suicide cases of the elderly can be observed more where they experience loneliness because of social ignorance.
